# The Effect of Anterior Capsule Polishing on Capsular Contraction and Lens Stability in Cataract Patients with High Myopia

**DOI:** 10.1155/2018/8676451

**Published:** 2018-10-23

**Authors:** Dandan Wang, Xiaoyu Yu, Zhangliang Li, Xixia Ding, Hengli Lian, Jianyang Mao, Yinying Zhao, Yun-E. Zhao

**Affiliations:** ^1^School of Optometry and Ophthalmology and Eye Hospital, Wenzhou Medical University, Wenzhou, Zhejiang, China; ^2^Key Laboratory of Vision Science, Ministry of Health P.R. China, Wenzhou, Zhejiang, China

## Abstract

**Purpose:**

To evaluate the effect of anterior capsule polishing in patients with high myopia after cataract surgery.

**Setting:**

The Eye Hospital of Wenzhou Medical University, Zhejiang, China.

**Design:**

Prospective study.

**Methods:**

High myopic patients with a bilateral cataract who underwent phacoemulsification with 360° anterior capsular polishing in one eye and without polishing in the contralateral eye were recruited. The following parameters were recorded at 1, 3, and 6 months postoperatively, including the area and diameter of the anterior capsule opening (area and D), IOL tilt and decentration, refraction, and postoperative aqueous depth (PAD).

**Results:**

Paired samples of 38 eyes of 19 patients were enrolled. The area decreased significantly in both the polished group and unpolished group, whereas the diameter reduced more in the unpolished group. The IOL tilt and decentration at 3-month and 6-month follow-up showed significant differences between two groups. In the unpolished group, the IOL decentration firstly appeared between one-month to three-month visit, while the refraction error, PAD, and IOL tilt were significantly different between the three-month and six-month visits.

**Conclusion:**

360° anterior capsule polishing can effectively reduce the extent of the anterior capsule contraction and increase the stability of IOL. The study was registered at http://www.clinicaltrials.gov, and the clinical trial accession number is NCT 03142269.

## 1. Introduction

Phacoemulsification has been referred to as a refractive surgery due to the development of surgical techniques and refraction-correcting IOLs. An accurate prediction of postoperative refraction is important in refractive cataract surgery. The anterior capsule contraction syndrome (ACCS) is a well-recognized postoperative complication. The outcomes of ACCS are opacification, fibrosis, and contraction of the anterior capsule [1–3]. In some previous studies, high myopia is the most common risk factor for advanced intracapsular IOL dislocation, with reported incidence ranging from19.7% to 40% [4, 5]. Several studies have reported that this is mostly observed under conditions of zonular weakness and chronic intraocular inflammation [6, 7].

High myopic patients, especially with axial length of >27.0 mm, probably have an enlarged capsular bag with weak zonules and tend to develop cataracts earlier than emmetropic patients [7, 8]. This increases the risk of postoperative intracapsular IOL dislocation and reduces the accuracy of predicting postoperative refractive error. Our previous study, using an ultralong-scan-depth OCT, suggested that weak capsular adhesion and incomplete adhesive capsular bend is increased in high myopic eyes [9]. These factors presumably increase the likelihood of capsular contraction and postoperative refraction deviation. A number of studies have reported that capsular contraction is associated with lens epithelial cell (LEC) proliferation and migration originating underneath the anterior capsule [10–12]. However, anterior capsule polishing remains controversial in the field [11–14]. To the best of our knowledge, no interocular comparison on anterior capsule opening contraction, tilt, and decentration of intraocular lenses in high myopic cataract patients after uneventful phacoemulsification surgery has been conducted. In each patient, we performed phacoemulsification with a 360° anterior capsular polishing in one eye, while the contralateral eye was left unpolished. The surgeon(Z.Y.E) designed an instrument to perform a 360° capsule polishing, featured like a paddle with thin blunt edge, and the tip is approximately 5.0 mm with a diameter of 1.0 mm.

The objective of this study is to determine if the anterior capsule polishing is beneficial to reduce the extent of the anterior capsule contraction and increase the stability of the intraocular lens.

## 2. Methods

### 2.1. Patients

This is a prospective patient-masked clinical trial. The study followed the tenets of the Helsinki agreement, and informed consent was obtained from all patients. In this study, 40 eyes from 20 patients were recruited in the Eye Hospital of Wenzhou Medical University. One patient was excluded because of a postoperative retinal tear and did not complete the follow-up protocol. The inclusion criteria included: cataract patients with extremely high myopia (axial length >27 mm); the difference between bilateral eyes was less than 1 mm; and age range from 50 to 75 years. Preoperative assessments included slit-lamp, dilated fundus examination by an ophthalmologist. The same examiner performed noncontact tonometry and optical biometry (IOLMaster 5.0, Carl Zeiss, Germany) measurements. Patients with keratitis, glaucoma, uveitis, retinitis pigmentosa, pseudoexfoliation syndrome, diabetes, or myotonic dystrophy were excluded. Additionally, patients with history of ophthalmic surgery, trauma, severe postoperative inflammatory reactions, or unsuccessful intraoperative continuous circular capsulorhexis (CCC) were excluded.

### 2.2. Surgical Technique

All surgeries were performed by the same experienced surgeon (Z. Y. E). Phacoemulsification (Alcon infiniti, USA) was performed and a one-piece hydrophilic, square-edged Akreos MI60 IOL with a total length of 11 mm and optical zone diameter length 6.2 mm (Bausch, USA) was implanted. After topical anesthesia, a 2.2 mm corneal incision was made. Ophthalmic viscosurgical devices (OVDs) were used to inflate the anterior chamber, and CCC (diameter range of 5.5 ± 0.2 mm) was performed, ensured the CCC size and shape to expected use of the digital navigation system. Hydrodissection and phacoemulsification were performed, and the cortex was removed with automated irrigation/aspiration. In the polished group, take the left eye for example, after the IOL implantation, a polisher was introduced into the chamber via a side port incision on the temporal to polish the superior and nasal quadrant of the inner surface of the anterior capsule and then via a main incision on the superior to polish the inferior and temporal quadrant capsule. In the unpolished group, the anterior capsule was left unpolished. All posterior capsules were polished.

According to the random number table, one eye was randomly assigned to the polished group, while the other eye was assigned to the unpolished group, and two operations were completed within one week. All cases of postoperative medication were standardized. Immediate application of eye drops occurred after surgery. Levofloxacin (0.5%, Santen, Japan) was administered four times a day for two weeks after surgery. Fluorometholone (0.1%, Santen, Japan) was administered four times a day for 4 weeks after surgery, and the dose was reduced once a week. Bromfenac sodium (0.1%, Stuton, Japan) was administered twice a day for six weeks after surgery.

### 2.3. Parameters

All patients were examined at one day, one week, and one, three, and six months after surgery. In every follow-up visit, BCVA and intraocular pressure were assessed. Fully dilated fundus exams were also performed. The refractive status and anterior capsule opening size were determined as baseline on the first day after operation. Measured parameters included the area and diameter of the anterior capsule opening (area and D), IOL tilt and decentration, refractive state, and postoperative aqueous depth (PAD). All exams were performed after pupil dilation with tropicamide phenylephrine (Santen, Japan). The investigator acquiring and measuring the data have been masked. Anterior-segmental photography using both diffused light and slit-light across the central visual axis was obtained from each patient. Image J software (2x, National Institutes of Health) was used to determine the anterior capsulorhexis size (Area and D).

**Figure 1:**
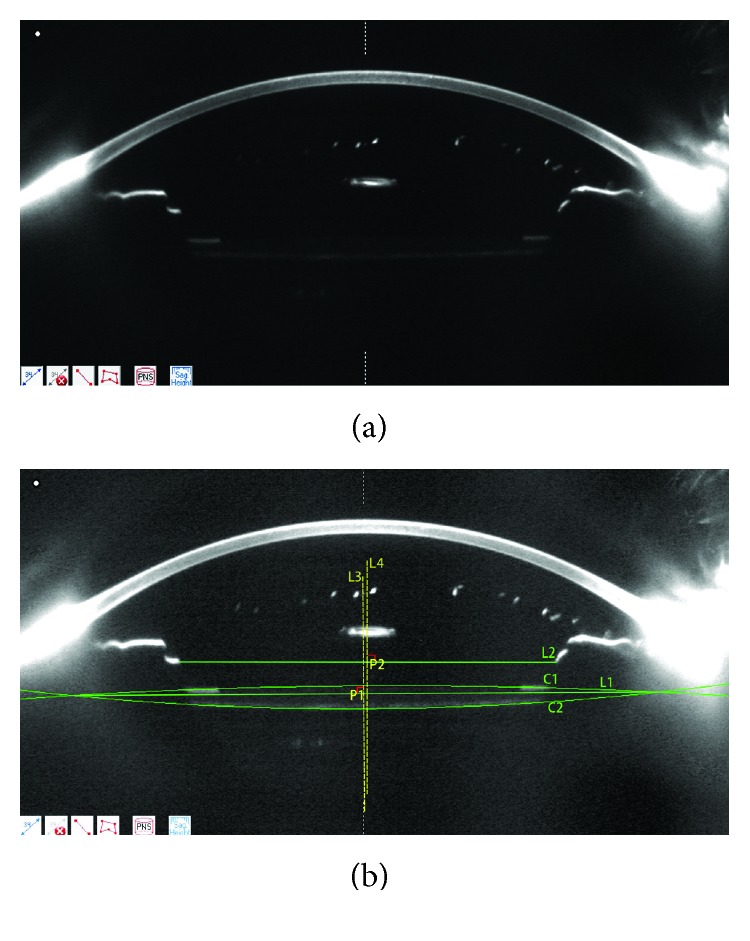
Anterior segment of an aphakic eye imaged by the Pentacam Scheimpflug imaging system. L3: axis on the optical center of IOL; L4: axis on the center of pupilla; C1: the best-fit-circle on the anterior surface of IOL; C2: the best-fit-circle on the posterior surface of IOL; L1: horizontal line of IOL; L2: horizontal line of pupilla. The tilt of IOL is defined as the angle between L3 and L4. The decentration of IOL is defined as the distance between horizontal coordinates of P1 and P2.

The commercially available Scheimpflug imaging system (Pentacam, Oculus) was used to measure the PAD, which is defined as the perpendicular distance from the central corneal endothelium to the anterior surface of IOL. The Pentacam Scheimpflug system also was used to measure IOL tilt and decentration [15, 16] (Figure 1). The pupillary axis was considered to be the center of pupil after mydriasis. Scheimpflug images at 90° and 180° from the pupillary axis were taken to get the best-fit-circle of the anterior and posterior surface of IOL to determine the center line of the IOL using Image Pro Plus(Media Cybernetics, Inc. USA). In order to determine the tilt of the IOL, using the pupillary axis as the baseline, the angle between the axis of optical center of IOL and the baseline was measured. Decentration of IOL was referred to as the difference between horizontal coordinates at the center of the IOL central line and the center of the pupillary axis. Differences were determined to be the change in mean values between examinations.

### 2.4. Statistical Analysis

Data analysis was performed using SPSS software for Windows (Version 19.0; SPSS Inc., Chicago, IL, U.S.). Kolmogorov–Smirnov tests were used to check the normal distribution of variables. Based on the design of experiment, paired *t*-tests were used for comparison between paired groups. And according to numeric results for paired *t*-tests calculated by SPSS software using the number of cases, parameters' means and standard deviation, the sample size was sufficient just need past 6 pairs, a sample of 19 cases was more than adequate for this study. The Wilcoxon rank test was used in cases that data did not have normal distribution. Nominal *p* values were calculated using *t* tests, and the level of significance was set at 0.05.

## 3. Results

A total of 38 eyes (19 patients) with mean age of 60.53 ± 10.18 years were included in this prospective study. No patients received Nd: YAG laser capsulotomy due to serious posterior capsule opacification (PCO) or CCS. There were no significant differences between the polished group and the unpolished group, in terms of the axial length (AL), refraction status, and anterior capsule opening size on the day after surgery (Table 1).

Although the IOL tilt did not show significant differences between groups at one month after operation (*p*=0.065), it was slightly lower in the polished group. And at the same time, the refraction status and anterior capsule opening size revealed no significant differences (Table 2). At the postoperative three-month visit, both the IOL tilt and decentration in the polished group were significantly lower than those in the unpolished group (*p*=0.047, *p*=0.045, respectively) (Table 3). And these differences were prolonged to the six-month follow-up (*p*=0.021 and 0.021, respectively) (Table 4). With respect to other parameters, no significant differences were found between two groups at both three-month and six-month follow-ups.

### 3.1. Longitudinal Comparison between Postoperative Time Points

#### 3.1.1. The Polished Group

**Figure 2:**
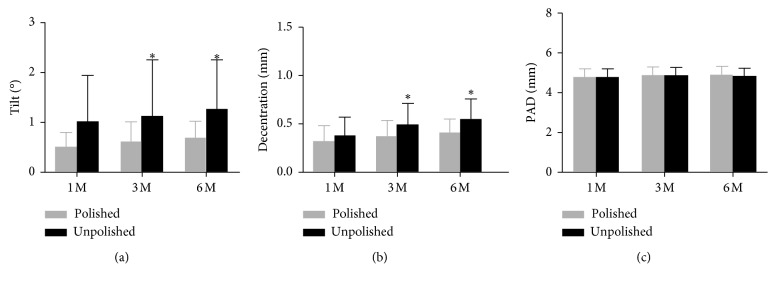
The differences in IOL stability between the two groups (^*∗*^Significant at *p* < 0.05).

**Figure 3:**
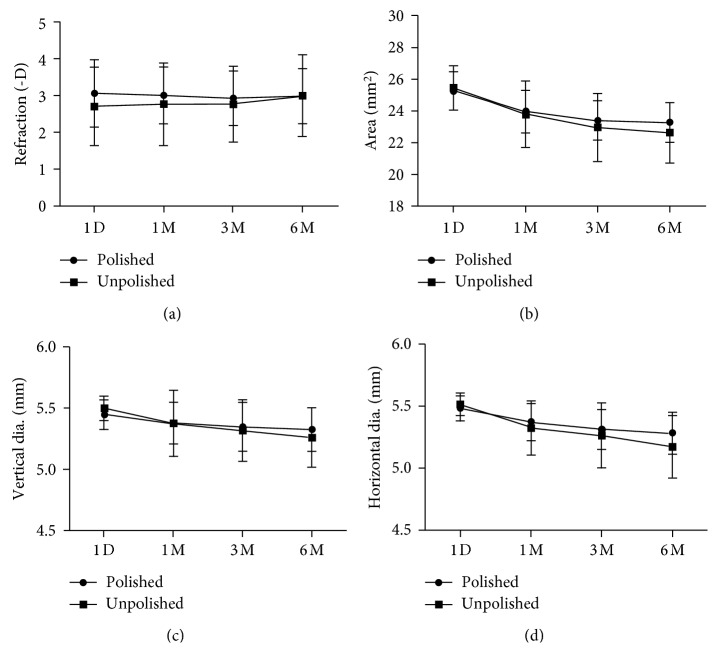
The differences in refraction and anterior capsule opening size between the two groups.

The anterior capsular opening area decreased significantly between each time points (all *p* < 0.001) while the horizontal diameter was significantly reduced (*p*=0.002, 0.017 and <0.001, respectively). The vertical diameter was significantly reduced only from the three-month visit to the six-month visit (*p*=0.004). The refraction error and PAD in the polished group showed no significant differences between different postoperative time points (Figures 2 and 3).

#### 3.1.2. The Unpolished Group

The anterior capsular opening area differed significantly between each time points (*p* < 0.001, <0.001, and 0.002, respectively). And the horizontal diameter was significantly reduced (*p* < 0.001, 0.099, and 0.005, respectively), while the vertical diameter reduced analogously (*p*=0.059, 0.004, and <0.001, respectively). The differences of refraction error and PAD in the unpolished group were significant between the three-month and six-month visits (*p*=0.011 and 0.003, respectively). The IOL tilt between three-month and six-month follow-ups was significantly different (*p*=0.045), while the IOL decentration between the one-month and three-month follow-ups was also different (*p*=0.018). (Figures 2 and 3).

## 4. Discussion

The risk of postoperative complications, such as ACCS, is increased in high myopic patients due to zonular weakness. This is caused by anterior capsular opening shrinkage, zonule elongation, or IOL tilt and decentration. In a previous retrospective analytical study, we demonstrated that the frequency of anterior capsular opening shrinkage and IOL tilt and decentration was significantly higher in cataract eyes with high myopia than that in cataract eyes with a normal axial length [17]. To our knowledge, no study with respect to how to effectively reduce the anterior capsular contraction has been published. Previous studies have reported that LECs play a major role in the pathogenesis of capsule contraction and fibrosis [1, 2, 4, 18]. To this end, we think it is important to ensure residual LECs were eliminated during the operation which can reduce the occurrence of anterior capsular contraction. However, the effectiveness of anterior capsule polishing during phacoemulsification still remains controversial [4,11–14,19]. Most ophthalmic surgeons do not perform anterior capsule polishing during phacoemulsification; however, many surgeons claim that anterior capsule polishing is beneficial. These advocates commonly use the I/A tip or other modified polisher through the main incision to polish the anterior capsule. However, the capsule at the main incision is particularly difficult to polish. With this understanding, we performed an interocular comparison of polished and unpolished capsules as it impacts anterior capsular contraction and intraocular lens stability in high myopic cataract patients. For this procedure, we used a newly designed polisher that is thin and smooth and has a tip that is approximately 5.0 mm long with a diameter of 1.0 mm. The improved instrument can be smoothly inserted through the second incision into the anterior chamber and be positioned 360° the inner surface of the anterior capsule.

No significant difference in the anterior capsule opening formation between the two groups was observed. However, we found the anterior capsule opening area of the two groups similarly contracted after the operation. This is due to myofibrillar contraction of LECs following transdifferentiation [20]. However, the vertical diameter in the polished group had a slight reduction in the early after surgery. Whereas, both vertical diameter and horizontal diameter showed more significant reduction in the unpolished group. Menapace and Di had proved anterior capsule polishing reduced capsulorhexis contraction, and the clinical results were similar by measuring the changes of the capsulorhexis opening [10]. This suggests that anterior capsule polishing can effectively reduce the contraction of anterior capsule and increase the stability of the intraocular lens.

The impact of IOL tilt and decentration on visual quality has been widespread recognized [21, 22]. Several studies suggest that more than 1 mm decentration and a greater than 5° tilt optically impairs visual quality. An average tilt and decentration of 3°and 0.25 mm, respectively, are well below the criteria to affect clinically observable visual acuity [22, 23]. In the present study, both the IOL tilt and decentration in the polished group showed no significant differences between different postoperative time points. By contrast, in the unpolished group, the IOL tilt between the three-month and six-month follow-ups was significantly different, while the IOL decentration between the one-month and three-month follow-ups was also significant different. We speculate that the IOL gradually tilted during the first month and appears statistically significant at three to six months in the unpolished group. The main reason for this is that myofibrillar contraction of the unpolished anterior capsule leads to tilt of the IOL.

In this study, using the Pentacam system to evaluate the PAD, we defined the PAD as the distance from the posterior surface of the corneal to the anterior IOL surface. This reflects the ELP, which has a clinically relevant impact on postoperative refraction [24]. These results indicate that the differences of refraction status and PAD in the unpolished group were significant between the three-month and six-month visits. During three to six months after surgery, the IOL gradually moved forward and the PAD decreased to a statistically significant degree. Due to the change of PAD, myopic shift occurred in 3–6 months after surgery in the unpolished group. This is consistent with the results of our previous retrospective study that showed PAD decreased in high myopic patients, as well as a decrease in the occurrence of a shallow anterior chamber and myopic shift. While the achieved refraction outcome and PAD in the polished group showed no significant differences between different postoperative time points. In conclusion, our results suggest that IOL placement is more stable in polished capsular bags.

Besides, during the six months follow-up even beyond the experiment, we observed no obvious difference in the incidence of PCO between polished eyes and unpolished eyes which was consistent with the results of the previous study of Liu et al. [14].

The limitation of this study is relatively small sample size and some differences, which has not reached any significant clinical impact in terms of patient symptoms. However, in the present study, all procedures were performed by the same surgeon, and the axial length of our subjects was limited to more than 27; the difference between bilateral eyes was less than 1 mm, and these may minimize influencing factors.

In conclusion, our study suggests that 360° anterior capsule polishing can effectively maintain stability of the position of the IOL-capsule complex.

## Figures and Tables

**Table 1:** The AL, refraction status, and anterior capsule opening size at the 1st day after surgery.

Parameter (*N*=19)	Polished	Unpolished	*t* value	*p*
AL (mm)	29.90 ± 1.68	29.99 ± 1.86	−0.544	0.583
Refraction status (D)	−3.05 ± 0.91	−2.70 ± 1.06	−1.347	0.195
Area (mm^2^)	25.26 ± 1.21	25.46 ± 1.40	−1.008	0.327
Vertical Dia.(mm)	5.45 ± 0.12	5.50 ± 0.10	−1.644	0.118
Horizontal dia. (mm)	5.48 ± 0.10	5.51 ± 0.09	−1.509	0.149

**Table 2:** Cross-sectional comparison between polished and unpolished groups at the 1 month after surgery.

Parameter (*N*=19)	Polished	Unpolished	*t* value	*p*
Refraction status (D)	−3.00 ± 0.77	−2.76 ± 1.12	−1.124	0.276
PAD (mm)	4.79 ± 0.39	4.79 ± 0.39	0.076	0.940
IOL tilt (°)	0.49 ± 0.30	1.06 ± 0.90	−0.364	0.065
IOL decentration (mm)	0.32 ± 0.16	0.38 ± 0.19	−0.973	0.344
Area (mm^2^)	23.97 ± 1.34	23.78 ± 2.11	0.495	0.626
Vertical dia. (mm)	5.38 ± 0.17	5.38 ± 0.27	0.026	0.979
Horizontal dia. (mm)	5.37 ± 0.15	5.32 ± 0.22	1.208	0.243

**Table 3:** Cross-sectional comparison between polished and unpolished groups at the 3 months after surgery.

Parameter (*N*=19)	Polished	Unpolished	*t* value	*p*
Refraction status (D)	−2.92 ± 0.74	−2.76 ± 1.03	−0.763	0.455
PAD (mm)	4.86 ± 0.41	4.87 ± 0.40	−0.138	0.891
IOL tilt (°)	0.61 ± 0.41	1.13 ± 1.02	−2.127	0.047^*∗*^
IOL decentration (mm)	0.37 ± 0.17	0.49 ± 0.22	−2.154	0.045^*∗*^
Area (mm^2^)	23.41 ± 1.24	22.97 ± 2.14	1.018	0.322
Vertical dia. (mm)	5.35 ± 0.20	5.32 ± 0.25	0.527	0.605
Horizontal dia.(mm)	5.31 ± 0.16	5.26 ± 0.26	0.773	0.450

^*∗*^Significant at *p* < 0.05.

**Table 4:** Cross-sectional comparison between polished and unpolished groups at the 6 months after surgery.

Parameter (*N*=19)	Polished	Unpolished	*t* value	*p*
Refraction status (D)	−2.97 ± 0.74	−2.99 ± 1.11	−0.127	0.900
PAD (mm)	4.87 ± 0.47	4.81 ± 0.42	0.645	0.527
IOL tilt (°)	0.69 ± 0.35	1.24 ± 1.00	−2.519	0.021^*∗*^
IOL decentration (mm)	0.42 ± 0.14	0.55 ± 0.21	−2.519	0.021^*∗*^
Area (mm^2^)	23.26 ± 1.24	22.64 ± 1.90	1.636	0.119
Vertical dia. (mm)	5.33 ± 0.18	5.26 ± 0.24	1.452	0.164
Horizontal dia.(mm)	5.28 ± 0.17	5.17 ± 0.25	1.563	0.135

^*∗*^Significant at *p* < 0.05.

## Additional Points

*What Was Known*: (i) The risk of postoperative complications, such as capsular contraction syndrome, is increased in high myopic patients due to zonular weakness. (ii) Cataract patients with highly myopia were more likely to experience anterior capsule contraction, particularly during the early postoperative period. (iii) The effectiveness of anterior capsule polishing during phacoemulsification still remains controversial. *What This Paper Adds*: (i) We reported the benefits of using 360° anterior capsule polishing, which can effectively reduce the extent of the anterior capsule contraction and increase the stability of the intraocular lens. (ii)It is worth considering performing 360° anterior capsule polishing during phacoemulsification in cataract patients with highly myopia.
